# Will artificial intelligence change medical life? Bridging the gap between innovation and medical student adoption: a cross-sectional study among university medical students

**DOI:** 10.3389/fmed.2026.1871352

**Published:** 2026-07-03

**Authors:** Mirfat Mohamed Labib Elkashif, Hebatalla Abdelmaksoud Abdelmonsef Ahmed, Mohamed Sayed Abdellatif, Darelglal Ahmed Gassmelseed, Shimaa Mohamed Mohamed Koabar

**Affiliations:** 1 Department of Nursing Sciences, College of Applied Medical Sciences in Wadi Aldawaser, Prince Sattam Bin Abdulaziz University, Al-Kharj, Wadi Aldawaser, Wadi Aldawaser, Saudi Arabia; 2 Department of Public Health and Community Medicine, Faculty of Medicine, Kafr-Elsheikh University, Kafr El-sheikh, Egypt; 3 Department of Psychology, College of Education in Al-Kharj, Prince Sattam Bin Abdulaziz University, Al-Kharj, Saudi Arabia; 4 Department of Public Health and Community Medicine, Faculty of Medicine, Tanta University, Tanta, Egypt

**Keywords:** artificial intelligence, medical students, attitude and practice, robotics technology adoption, clinical decision support systems

## Abstract

**Background:**

Artificial intelligence and robotics are transforming healthcare by improving diagnosis, treatment, and personalized medicine. Understanding medical students’ and faculty perceptions is essential for effective integration into medical education. This study examines the benefits, limitations, and impact of AI on clinical practice, education, and healthcare delivery. This research investigates the role of artificial intelligence (AI) in medicine, highlighting its advantages and limitations, with emphasis on its applications in various clinical fields and its influence on medical education and healthcare delivery.

**Methods:**

Across-sectional study was conducted involving 441 medical students at Tanta University. Data was gathered by using a structured pretested self-administered questionnaire, developed from the literature, and validated by experts. It covered sociodemographic characteristics, perceptions toward AI in medical education, and knowledge and attitudes regarding AI use in healthcare.

**Results:**

The survey revealed that 95.9% of participants have heard about AI, primarily through media (77.8%). with 76.0% showing a favorable attitude. Most students (58.3%) reported learning about AI through online communities and forums. However, only 37.9% learned AI techniques through courses or training. Less than one quarter (23.8%) consider Chatbot for student support the most useful. The most frequently reported benefit was its ability to speed up the management process (46.3%), followed by its capacity to capture and analyze more information than humans (42.9%) and its potential to reduce medical errors (42.9%). Students raised concerns about reduced independent thinking (52.6%) and limited interpersonal skills (47.4%). The main barriers identified were lack of proper training (51.9%), limited financial resources (49%), and lack of awareness (47.6%).

**Conclusion:**

Medical students showed high awareness and positive attitudes toward AI but had limited formal training. They recognized its benefits in learning and clinical practice while expressing ethical concerns regarding ethics, reduced human interaction, and accountability. These findings highlight the need to incorporate structured AI education into medical curricula, emphasizing both technical competencies and ethical frameworks, to prepare future physicians for safe and effective AI adoption in healthcare.

## Introduction

Artificial intelligence (AI), first defined by John McCarthy in 1955, refers to the capacity of machines to simulate human reasoning and actions, though not in the same way humans think or behave (1, 2). The use of AI in medicine began in the 1950s, with early computer-assisted diagnostic programs such as Gunn’s 1976 abdominal pain analysis (3–5).

Advances in computing power have since fueled rapid growth, with AI now embedded in nearly 77% of devices and a marked rise in AI-related startups (5). Globally, adoption of AI in healthcare is more advanced in developed countries, while developing regions, including Egypt, face challenges related to infrastructure and resources. However, ongoing initiatives aim to expand AI integration and improve access to its medical applications (5).

Artificial Intelligence (AI) is rapidly advancing across multiple industries, with medicine being one of the most impacted. It has shown transformative potential in areas such as diagnosis, treatment planning, personalized medicine, and drug discovery. AI already plays a significant role in specialties like radiology, neurosurgical imaging, dermatology, oncology, cardiology, and neurology, including early detection of diseases such as Alzheimer’s and breast cancer (1, 2). Given its expanding applications, healthcare professionals must understand both the benefits and limitations of AI, as they will increasingly participate in its development and integration into clinical practice (1). Consequently, AI is being incorporated into undergraduate and postgraduate medical education, where it provides targeted feedback and helps students better grasp algorithmic decision-making (2).

The integration of artificial intelligence (AI) and machine learning (ML) into healthcare is often hindered by limited physician knowledge, which may negatively affect patient outcomes. This gap stems from the minimal coverage of AI in undergraduate medical curricula, the absence of accreditation requirements, overloaded course content, lack of qualified faculty, and uncertainty about the physician’s evolving role alongside AI (1). Studies reveal that both clinicians and students exhibit varying degrees of awareness. For example, a Korean study found that while many physicians were unfamiliar with AI, they still acknowledge its benefits in medical practice (4). Similarly, medical students generally agree that AI will advance medicine but reject the idea that it will replace doctors in the near future (3).

However, concerns persist that AI could threaten certain specialties, discouraging students from pursuing them as career paths (3).

The development of AI curricula in medical education requires close collaboration among data scientists, computer scientists, clinicians, and medical educators. Since AI covers diverse skills and subject areas, a range of teaching and learning strategies is needed to ensure students gain a practical understanding of AI concepts for use in patient care (1). Raising awareness early is essential to avoid misconceptions about AI in medicine and to prepare future physicians for effective application of these technologies. Medical training should therefore extend beyond biological and clinical sciences to include competencies necessary for employing AI systems, particularly given their growing role in healthcare. The Association of American Medical Colleges (AAMC) has long recommended the use of simulation technologies, underscoring the need for medical education to shift from the information age toward the AI era, where machine learning supports clinical decision-making (4). Recent work illustrates how AI systems can utilize structured datasets such as ICD-10, which include disease categories, symptoms, causes, and locations, to generate health indicators based on user input queries. Such approaches demonstrate the potential of AI to enhance clinical decision support and improve patient care (3, 6).

With the rapid integration of AI and robotics into healthcare, future medical professionals will inevitably encounter these technologies (7–10).

To prepare students for this transition, faculty must adopt and incorporate such innovations into medical education. Currently, little research has explored the perspectives of both medical students and faculty regarding AI and robotics in medicine. This study aims to address that gap by evaluating their views on the importance of these technologies and their potential influence on the future of healthcare.

## Methods

### Study design, setting, and participants

A cross-sectional study was conducted among undergraduate medical students and medical interns at the Faculty of Medicine, Tanta University, Egypt. The study included students from the first through fifth academic years as well as medical interns. Data were collected over a five-month period from November 2024 to March 2025.

### Sample size and sampling technique

A non-probability convenience sampling technique was employed. The target population consisted of approximately 8,000 undergraduate medical students and interns enrolled at the Faculty of Medicine, Tanta University.

The minimum required sample size was calculated using Epi Info™ version 7.2.3.0 (Centers for Disease Control and Prevention, Atlanta, GA, USA). Based on a previously reported awareness level of artificial intelligence (AI) among Egyptian medical students of 65.4%, a 95% confidence level, and a precision of ±5%, the minimum required sample size was estimated to be 329 participants. To compensate for potential non-response and incomplete questionnaires, the sample size was increased to 441 participants (7, 11).

### Eligibility criteria

#### Inclusion criteria

The study included undergraduate medical students from the first through fifth academic years and medical interns who:

- Were available during the data collection period.
- Had prior exposure to artificial intelligence concepts in medical education or healthcare settings.
- Agreed voluntarily to participate in the study.

#### Exclusion criteria

Students were excluded if they:

- Were absent or on vacation during the study period.
- Were hospitalized or suffering from serious illness during data collection.
- Declined participation.

#### Participant recruitment and data collection

Participants were recruited using convenience sampling from all academic years and internship levels. Eligible students were approached during academic activities and informed about the study objectives and procedures. Written informed consent was obtained prior to participation.

Data were collected using an anonymous, structured, self-administered questionnaire. Participants completed the questionnaire independently, and completed forms were checked for completeness before data entry and analysis. Confidentiality and anonymity were assured throughout the study.

#### Study instrument

Data were collected using a structured, pretested, self-administered questionnaire developed after an extensive review of the relevant literature (7, 11).

The questionnaire consisted of 32 items divided into three sections:

### Section I: sociodemographic characteristics

This section included seven items assessing participants’ age, sex, academic year, residence, family income, marital status, and prior awareness of artificial intelligence.

### Section II: perception of artificial intelligence in medical education

This section assessed students’ perceptions of AI integration in medical education, learning readiness, perceived benefits, and barriers to implementation.

### Section III: knowledge and attitudes regarding artificial intelligence in healthcare

This section consisted of 15 items assessing knowledge and perceptions regarding AI applications in healthcare, including intended clinical use, expected timeline for integration into practice, privacy concerns, ethical considerations, and perceived advantages of AI technologies.

### Questionnaire scoring

Attitudes toward artificial intelligence in medical education were assessed using five items (Q6–Q10). Responses were scored as:

- Agree = 2
- Disagree = 1

A total attitude score was calculated by summing individual item scores and converting the result into a percentage score according to the following formula:


Percentage score=(Obtained score/Maximum possible score)×100

Participants scoring ≥60% were categorized as having a positive attitude toward AI, whereas those scoring <60% were categorized as having a negative attitude.

### Validity and reliability

Content validity was assessed by three experts from the Public Health and Community Medicine Department. Experts evaluated each item using a four-point relevance scale ranging from 1 (not relevant) to 4 (completely relevant).

The Content Validity Index (CVI) was calculated by dividing the number of experts rating an item as either 3 or 4 by the total number of experts. Items with a CVI ≥ 0.80 were considered acceptable.

The initial questionnaire contained 34 items. Following an expert review, two items were removed, resulting in a final 32-item instrument. All remaining items achieved acceptable content validity, yielding an overall CVI of 1.0.

Internal consistency reliability was assessed using Cronbach’s alpha coefficient, which demonstrated good reliability (*α* = 0.86).

### Pilot study

A pilot study was conducted on 40 medical students (approximately 10% of the calculated sample size) who were not included in the final analysis. The pilot study evaluated the clarity, feasibility, applicability, and completion time of the questionnaire.

Based on participant feedback and pilot study findings, minor modifications were made to improve clarity and comprehensibility before the main data collection phase.

### Ethical considerations

The study was conducted in accordance with the principles of the Declaration of Helsinki.

Ethical approval was obtained from the Research Ethics Committee of the Faculty of Medicine, Tanta University (Approval No. 36264PR493/1/24). Written informed consent was obtained from all participants before enrollment. Participation was voluntary, and participants were assured of the confidentiality and anonymity of their responses. Data were used solely for research purposes.

### Statistical analysis

Data were coded, entered, cleaned, and analyzed using the Statistical Package for Social Sciences (SPSS), version 22.0 (IBM Corp., Armonk, NY, USA).

Descriptive statistics were used to summarize study variables. Categorical variables were presented as frequencies and percentages, whereas continuous variables were expressed as mean ± standard deviation (SD).

Associations between categorical variables were examined using the Chi-square (χ^2^) test. When expected cell frequencies were less than five, the Monte Carlo exact test was applied.

The reliability of the study instrument was evaluated using Cronbach’s alpha coefficient, and content validity was assessed using the Content Validity Index.

All statistical tests were two-tailed, and a *p*-value <0.05 was considered statistically significant.

## Results

The study included 441 medical students with a mean age of 22.0 ± 1.0 years, predominantly male (61.9%), and most were in advanced academic years, with half (50.8%) in the 5th year. Most students resided in urban areas (59.4%), and nearly all participants were single (95.0%). Awareness of AI was high, with 95.9% reporting having heard about AI, although only 13.4% had heard about AI-related courses, and a mere 6.6% had received formal AI training.

Most students relied on informal sources for AI information, particularly media (77.8%) and friends/family (29.7%), while formal sources like TV (19.3%) and structured training (6.6%) were less common. This highlights a significant gap in structured AI education (Table 1).

**Table 1:** Sociodemographic characteristics and awareness of artificial intelligence (AI) among the studied students (*n* = 441).

Studied variables		*N* = 441	%
Age (Years): *Mean ± SD*		22.0 ± 1.0	
Gender	Male	273	61.9%
	Female	168	38.1%
Academic year	1st year	18	4.1%
	2nd year	38	8.6%
	3rd year	52	11.8%
	4th year	84	19.0%
	5th year	224	50.8%
	6th year	25	5.7%
Residence	Urban	262	59.4%
	Rural	179	40.6%
Marital status	Single	419	95.0%
	Married	22	5.0%
Hearing about AI before	Yes	423	95.9%
Hearing about AI courses	Yes	59	13.4%
Sources of AI Information	Received training	29	6.6%
	Media	343	77.8%
	TV	85	19.3%
	Friends/family	131	29.7%
Attitude towards AI	Positive attitude	335	76.0%
	Negative attitude	106	24.0%

Appendix: positive attitude > = 60%/Negative attitude <60%.

Attitudes toward AI were predominantly positive, with 76.0% showing a favorable attitude. This reflects growing acceptance of AI technologies among medical students, consistent with global studies showing that younger healthcare professionals tend to embrace AI as a tool for improving efficiency and decision-making when adequate awareness is present (Table 2).

**Table 2:** Students’ perspectives on learning artificial intelligence (AI) and its integration into medical education (*n* = 441).

Studied variables		*N* = 441	%
How students learn AI	Courses training	167	37.9%
	Curriculum	122	27.7%
	Online communities and forums	257	58.3%
Useful AI tools in medical education	Chatbots	105	23.8%
	Automated exam grading system	90	20.4%
	Virtual patient learning	101	22.9%
	Interactive smart boards	77	17.5%
Suggested AI topics to be included in the curriculum	Radiology and digital imaging	260	59.0%
	Disease prediction models	139	31.5%
	Medical genetics and genomics	177	40.1%
	Clinical trials	170	38.5%
	Precision medicine and new drug development	136	30.8%
	Diagnostics and clinical decision support	145	32.9%
	Individualize health data/device monitoring	121	27.4%
Disadvantages of AI in medical education	Lack of independent thinking	232	52.6%
	Limited exposure to difficult cases	198	44.9%
	Ethical considerations	201	45.6%
	Limited interpersonal skills	209	47.4%
Causes of AI implementation failure	Lack of interest	126	28.6%
	Lack of awareness	210	47.6%
	Lack of proper training	229	51.9%
	Lack of curriculum	90	20.4%
	Lack of financial resources	216	49.0%
	Lack of technological advancement	211	47.8%

As for the sources of information, the majority of students (58.3%) reported learning about AI through online communities and forums. This reflects the global trend toward self-directed, internet-based learning, driven by the limited integration of AI topics within formal medical curricula. Only 37.9% indicated learning through formal courses and 27.7% via curriculum-based content, highlighting a clear gap in structured AI education.

Regarding useful AI tools in medical education, students identified chatbots (23.8%), virtual patient learning (22.9%), and automated exam grading systems (20.4%) as the most beneficial. These preferences suggest that students value AI applications offering interactive and personalized learning experiences, consistent with global evidence that emphasizes AI’s role in enhancing clinical training and assessment.

Concerning suggested AI topics for curriculum integration, a significant proportion of students (59%) prioritized radiology and digital imaging, followed by medical genetics and genomics (40.1%) and clinical trials (38.5%). These findings align with international trends in which imaging and precision medicine are regarded as areas with the greatest potential for AI impact.

With respect to disadvantages of AI in medical education, students raised concerns about reduced independent thinking (52.6%) and limited interpersonal skills (47.4%), echoing global ethical debates on AI’s potential to diminish critical reasoning and doctor–patient interaction. Ethical considerations were also cited by 45.6% of participants as a major concern.

Finally, regarding causes of AI implementation failure, the main barriers identified were lack of proper training (51.9%), limited financial resources (49%), and lack of awareness (47.6%). These results are consistent with global reports indicating that insufficient training and inadequate infrastructure remain the primary obstacles to effective AI adoption in medical education.

The analysis reveals that 76% of medical students demonstrated a positive attitude towards AI, while 24% had a negative attitude. Among the studied factors, academic year showed a significant association with students’ attitudes towards AI (*p* = 0.001), with second-year students exhibiting the highest positive attitude (92.1%), while fourth-year students had the lowest (61.9%). No significant differences in AI attitude were observed with respect to gender, residence, marital status, or prior awareness of AI (*p* > 0.05). Overall, 76% of students demonstrated a positive attitude towards AI integration in medical practice (Table 3).

**Table 3:** Association between demographic variables and attitudes toward artificial intelligence in healthcare (*N* = 441).

Variable	Category	Positive attitude 335 (76.0%)	Negative attitude 106 (24.0%)	Total 441 (100%)	Test
					*p*-value
Gender	Male	202 (74.0)	71 (26.0)	273 (61.9)	*χ*^2^ = 1.525
	Female	133 (79.2)	35 (20.8)	168 (38.1)	*p* = 0.217
Academic year	Year 1	13 (72.2)	5 (27.8)	18 (4.1)	Mc = 20.159
	Year 2	35 (92.1)	3 (7.9)	38 (8.6)	*p* = 0.001*
	Year 3	45 (86.5)	7 (13.5)	52 (11.8)	
	Year 4	52 (61.9)	32 (38.1)	84 (19.0)	
	Year 5	174 (77.7)	50 (22.3)	224 (50.8)	
	Internship/Residency	16 (64.0)	9 (36.0)	25 (5.7)	
Residence	Urban	195 (74.4)	67 (25.6)	262 (59.4)	*χ*^2^ = 0.834
	Rural	140 (78.2)	39 (21.8)	179 (40.6)	*p* = 0.361
Marital status	Single	320 (76.4)	99 (23.6)	419 (95.0)	Mc = 2.694
	Married	12 (63.2)	7 (36.8)	19 (4.3)	*p* = 0.441
	Separated	1 (100.0)	0 (0.0)	1 (0.2)	
	Divorced	2 (100.0)	0 (0.0)	2 (0.5)	
Heard about AI before	No	13 (72.2)	5 (27.8)	18 (4.1)	*χ*^2^ = 0.144
	Yes	322 (76.1)	101 (23.9)	423 (95.9)	*p* = 0.704

X^2^, Chi-square; MC, Montecarlo. *Means significant.

As regarding the advantages of using AI in healthcare, we found that the most frequently reported benefit was its ability to speed up the management process (46.3%), followed by its capacity to capture and analyze more information than humans (42.9%) and its potential to reduce medical errors (42.9%). Concerning the impact of AI on patient outcomes and care quality, the highest proportion of students highlighted its role in monitoring chronic conditions (51.1%) and early disease detection (49.8%). With respect to willingness to interact with AI systems, nearly half of the participants (47.6%) were willing to use AI chatbots to answer health-related questions, while 41.0% accepted using them for arranging appointments. Regarding attitudes toward technology, the most common concern expressed was that patient contact might suffer (36.9%). In terms of liability for AI-related mistakes, more than half of the respondents (52.4%) believed that the company that created the AI should be responsible. As for privacy issues, the main concern raised was the sharing of data between parties and its illegal use (56.0%). When considering the expected problems of AI, the majority reported that AI is not flexible enough to be applied to every patient (58.9%). With regard to specialties most likely to benefit from AI, clinical fields involving image analysis such as radiology, pathology, and dermatology were most frequently reported (57.6%). Concerning useful AI tools in medical practice, electronic health records were the top response (48.9%), followed by screening tools (47.0%). Finally, regarding the perceived capabilities of AI, the majority indicated that AI could best support physicians in diagnosis (57.8%) and contribute significantly to drug research and development (51.5%) (Table 4).

**Table 4:** Participants’ perceptions and attitudes toward the use of artificial intelligence (AI) in healthcare (*n* = 441).

Item	*n* (%)
Advantages of AI in healthcare	
AI can capture and analyze significantly more information than humans	189 (42.9)
AI can make diagnoses faster and more accurately	150 (34.0)
Cost reduction	101 (22.9)
AI can speed up healthcare management processes	204 (46.3)
AI can reduce the number of medical errors	189 (42.9)
AI can deliver clinically relevant, high-quality data in real time	106 (24.0)
AI has no emotional exhaustion or physical limitations	139 (31.5)
Giving physicians more time for discussions and clinical examinations	138 (31.3)
Reduction in the number of additional examinations	108 (24.5)
Reduction in patient anxiety and greater patient participation in healthcare	70 (15.9)
Impact of AI on patient outcomes and care quality	
AI helps in early disease detection	219 (49.8)
AI helps in monitoring chronic conditions	225 (51.1)
AI helps in predicting health deterioration	183 (41.6)
AI improves patient outcomes and overall care quality	190 (43.2)
AI-driven tools enable minimally invasive procedures and faster recovery	165 (37.5)
Willingness to interact with AI systems	
AI chatbots answering health-related questions	209 (47.6)
AI providing diagnoses about health status	146 (33.3)
AI suggesting treatments	151 (34.4)
AI carrying out tests or examinations	163 (37.1)
AI arranging medical appointments	180 (41.0)
AI providing support and additional health information	111 (25.3)
General attitudes toward technology	
Fear of increased monitoring at work	123 (28.0)
Concern that patient contact will suffer	162 (36.9)
Concern that works will be less valued	143 (32.6)
Fear of losing job due to AI	106 (24.1)
Fear of reduced autonomy at work	144 (32.8)
Concern that qualifications may become insufficient	141 (32.1)
Concern that income may be negatively affected	135 (30.8)
Liability for AI errors	
Physician responsible for patient care	177 (40.9)
Company that created the AI	227 (52.4)
Patient who consented to follow AI advice	132 (30.5)
Independent AI system	74 (17.1)
Privacy concerns related to AI	
Facial and voice recognition technologies	165 (37.4)
Collection and storage of patients’ personal medical data	159 (36.1)
Sharing data between parties and illegal data use	247 (56.0)
Disclosure of sensitive personal data	156 (35.4)
Security threats such as hacking or data theft	176 (39.9)
Expected problems with AI	
Inability to provide opinions in unexpected situations due to insufficient information	197 (44.8)
Not flexible enough to be applied to every patient	259 (58.9)
Increased healthcare costs	120 (27.3)
Does not consider patients’ emotional wellbeing	164 (37.3)
Errors in diagnosis or management	139 (31.6)
Misuse of patient information by employers or health companies	160 (36.4)
Physicians may rely too much on AI and lose medical skills	171 (38.9)
May undermine physician and patient autonomy	85 (19.3)
AI developed by programmers with limited medical experience	138 (31.4)
Specialties expected to use AI most	
Image-based specialties (radiology, pathology, dermatology)	253 (57.6)
Surgical procedures	215 (49.0)
Public primary care	148 (33.7)
Other	33 (7.5)
Useful AI tools in medical practice	
Electronic health records	215 (48.9)
Clinical decision support systems	131 (29.8)
Medical imaging analysis	204 (46.4)
Preventive tools (health apps and wearables)	130 (29.5)
Digital screening and risk profiling	207 (47.0)
Diagnostic tools (e.g., analysis of X-rays or images)	135 (30.7)
Personalized medicine	117 (26.6)
AI-assisted therapy (robot-assisted surgery)	134 (30.5)
Patient monitoring and data management	105 (23.9)
Potential roles of AI in healthcare	
Supporting physicians in making diagnoses	255 (57.8)
Supporting treatment decisions	196 (44.4)
Supporting physicians during treatment	130 (29.5)
Patients managing their health independently using AI	105 (23.8)
Improving drug research and development	227 (51.5)
Supporting personalized medicine	161 (36.5)

Regarding the intention to use AI in daily practice, the majority of students reported they would use it “sometimes” (55.6%), with a higher proportion among males (61.2%) compared to females (38.8%). This trend was most notable among 5th-year students (50.6%). Only 2.9% indicated they would “always” use AI, with a higher proportion again among males (76.9%). Concerning the effect of AI on the number of patient appointments, most students reported they “do not know” (31.1%), while 27.0% believed AI would decrease appointments, and 23.8% felt it would increase them. This uncertainty was especially common among 5th-year students (48.9%) (Table 5).

**Table 5:** Students’ perceptions of artificial intelligence (AI) in medical practice according to gender and academic year (*n* = 441).

Response	Male *n* (%)	Female *n* (%)	Total *n* (%)	*p*-value	Year 1 *n* (%)	Year 2 *n* (%)	Year 3 *n* (%)	Year 4 *n* (%)	Year 5 *n* (%)	Residency *n* (%)	Total *n* (%)	M C/*p*-value
Intention to use AI in daily practice												
Never	20 (64.5)	11 (35.5)	31 (7.0)	5.369	0 (0.0)	1 (3.2)	4 (12.9)	6 (19.4)	20 (64.5)	0 (0.0)	31 (7.0)	29.563
Rarely	42 (70.0)	18 (30.0)	60 (13.6)	0.373	6 (10.0)	3 (5.0)	11 (18.3)	11 (18.3)	25 (41.7)	4 (6.7)	60 (13.6)	0.241
Sometimes	150 (61.2)	95 (38.8)	245 (55.6)		9 (3.7)	27 (11.0)	23 (9.4)	46 (18.8)	124 (50.6)	16 (6.5)	245 (55.6)	
Frequently	36 (52.9)	32 (47.1)	68 (15.4)		1 (1.5)	4 (5.9)	11 (16.2)	11 (16.2)	38 (55.9)	3 (4.4)	68 (15.4)	
Most of the time	15 (62.5)	9 (37.5)	24 (5.4)		2 (8.3)	3 (12.5)	1 (4.2)	7 (29.2)	9 (37.5)	2 (8.3)	24 (5.4)	
Always	10 (76.9)	3 (23.1)	13 (2.9)		0 (0.0)	0 (0.0)	2 (15.4)	3 (23.1)	8 (61.5)	0 (0.0)	13 (2.9)	
AI effect on number of patient appointments												
Increases	66 (62.9)	39 (37.1)	105 (23.8)	4.301	5 (4.8)	13 (12.4)	16 (15.2)	23 (21.9)	43 (41.0)	5 (4.8)	105 (23.8)	17.323
Decreases	71 (59.7)	48 (40.3)	119 (27.0)	0.231	5 (4.2)	4 (3.4)	10 (8.4)	20 (16.8)	74 (62.2)	6 (5.0)	119 (27.0)	0.300
Not alters	57 (71.3)	23 (28.7)	80 (18.1)		4 (5.0)	6 (7.5)	12 (15.0)	13 (16.3)	40 (50.0)	5 (6.3)	80 (18.1)	
Do not know	79 (57.7)	58 (42.3)	137 (31.1)		4 (2.9)	15 (10.9)	14 (10.2)	28 (20.4)	67 (48.9)	9 (6.6)	137 (31.1)	
Time needed for AI to become routine in practice												
>20 years	63 (63.6)	36 (36.4)	99 (22.4)	2.069	4 (4.0)	12 (12.1)	17 (17.2)	25 (25.3)	37 (37.4)	4 (4.0)	99 (22.4)	30.167
2–15 years	69 (61.6)	43 (38.4)	112 (25.4)	0.723	6 (5.4)	6 (5.4)	12 (10.7)	18 (16.1)	64 (57.1)	6 (5.4)	112 (25.4)	0.067
3–10 years	79 (57.7)	58 (42.3)	137 (31.1)		3 (2.2)	11 (8.0)	10 (7.3)	25 (18.2)	81 (59.1)	7 (5.1)	137 (31.1)	
4–5 years	50 (66.7)	25 (33.3)	75 (17.0)		4 (5.3)	8 (10.7)	12 (16.0)	14 (18.7)	29 (38.7)	8 (10.7)	75 (17.0)	
Never	12 (66.7)	6 (33.3)	18 (4.1)		1 (5.6)	1 (5.6)	1 (5.6)	2 (11.1)	13 (72.2)	0 (0.0)	18 (4.1)	
Concern that AI may replace specialists												
Doctors lose positions	62 (66.7)	31 (33.3)	93 (21.1)	2.272	3 (3.2)	6 (6.5)	9 (9.7)	24 (25.8)	46 (49.5)	5 (5.4)	93 (21.1)	20.521
Fewer doctors trained	75 (59.1)	52 (40.9)	127 (28.8)	0.518	6 (4.7)	12 (9.4)	21 (16.5)	22 (17.3)	63 (49.6)	3 (2.4)	127 (28.8)	0.153
No effect on job market	65 (65.0)	35 (35.0)	100 (22.7)		2 (2.0)	4 (4.0)	9 (9.0)	20 (20.0)	57 (57.0)	8 (8.0)	100 (22.7)	
AI creates new jobs	71 (58.7)	50 (41.3)	121 (27.4)		7 (5.8)	16 (13.2)	13 (10.7)	18 (14.9)	58 (47.9)	9 (7.4)	121 (27.4)	
If physician and AI disagree												
Physician opinion	159 (61.2)	101 (38.8)	260 (59.0)	3.370	9 (3.5)	20 (7.7)	28 (10.8)	47 (18.1)	142 (54.6)	14 (5.4)	260 (59.0)	11.595
AI opinion	42 (72.4)	16 (27.6)	58 (13.2)	0.185	3 (5.2)	6 (10.3)	12 (20.7)	13 (22.4)	23 (39.7)	1 (1.7)	58 (13.2)	0.313
Patient choice	72 (58.5)	51 (41.5)	123 (27.9)		6 (4.9)	12 (9.8)	12 (9.8)	24 (19.5)	59 (48.0)	10 (8.1)	123 (27.9)	

In terms of the expected timeframe for AI to become a routine tool in medical practice, the largest proportion anticipated this within 3–10 years (31.1%), followed by 2–15 years (25.4%). Male students were slightly more optimistic, with 57.7% expecting integration within 3–10 years compared to 42.3% of females. Academic year analysis showed that expectations for AI adoption were again highest among 5th-year students (59.1%).

When asked about AI’s effect on the physician job market, 28.8% believed fewer doctors would be trained in certain professions, while 27.4% thought AI would create new positions. A smaller proportion (22.7%) felt AI would not affect the job market. Male respondents more frequently believed in job loss (66.7%), while females were more likely to anticipate new job creation (41.3%). Finally, in the case of disagreement between physician and AI, the majority (59.0%) favored the physician’s opinion, with males (61.2%) more supportive than females (38.8%). Academic year distribution showed that this was most prominent among 5th-year students (54.6%). Meanwhile, 27.9% preferred to follow the patient’s choice, and only 13.2% supported AI’s judgment.

Regarding students’ perceptions of AI in assessment and education, the majority (77.1%) agreed they would feel more objectively graded if AI systems replaced standardized preceptors. This perception was more common among males (63.8%) than females (36.2%) and was especially prevalent among 5th-year students (51.2%). Similarly, more than half of the students (54.9%) agreed that AI assessment during clinical skills exams would provide more personalized feedback than human standardized patients, with the highest agreement observed among 5th-year students (48.8%) (Table 6).

**Table 6:** Students’ perceptions of AI in medical education according to gender and academic year (*n* = 441).

Response	Male *n* (%)	Female *n* (%)	Total *n* (%)	X2/*p*-value	Year 1 *n* (%)	Year 2 *n* (%)	Year 3 *n* (%)	Year 4 *n* (%)	Year 5 *n* (%)	Residency	Total *n* (%)	MC/*p*-value
Q13—Feeling more objectively assessed if AI grades instead of standardized preceptor												
Agree	217 (63.8)	123 (36.2)	340 (77.1)	2.318	16 (4.7)	25 (7.4)	42 (12.4)	65 (19.1)	174 (51.2)	18 (5.3)	340 (77.1)	4.981
Disagree	56 (55.4)	45 (44.6)	101 (22.9)	0.128	2 (2.0)	13 (12.9)	10 (9.9)	19 (18.8)	50 (49.5)	7 (6.9)	101 (22.9)	0.418
Q14—Personalized feedback from AI vs. human standardized patient												
Agree	157 (64.9)	85 (35.1)	242 (54.9)	2.008	12 (5.0)	15 (6.2)	30 (12.4)	51 (21.1)	118 (48.8)	16 (6.6)	242 (54.9)	7.251
Disagree	116 (58.3)	83 (41.7)	199 (45.1)	0.157	6 (3.0)	23 (11.6)	22 (11.1)	33 (16.6)	106 (53.3)	9 (4.5)	199 (45.1)	0.203
Q15—Using AI simulator instead of patients in clinical rounds												
Agree	150 (65.2)	80 (34.8)	230 (52.2)	2.237	12 (5.2)	14 (6.1)	22 (9.6)	49 (21.3)	115 (50.0)	18 (7.8)	230 (52.2)	12.401
Disagree	123 (58.3)	88 (41.7)	211 (47.8)	0.135	6 (2.8)	24 (11.4)	30 (14.2)	35 (16.6)	109 (51.7)	7 (3.3)	211 (47.8)	0.030*
Q16—Prefer personalized AI-generated questions vs traditional questions												
Agree	169 (63.8)	96 (36.2)	265 (60.1)	0.983	10 (3.8)	16 (6.0)	33 (12.5)	55 (20.8)	135 (50.9)	16 (6.0)	265 (60.1)	6.704
Disagree	104 (59.1)	72 (40.9)	176 (39.9)	0.321	8 (4.5)	22 (12.5)	19 (10.8)	29 (16.5)	89 (50.6)	9 (5.1)	176 (39.9)	0.244
Q17—AI can replace traditional teaching methods												
Agree	151 (64.3)	84 (35.7)	235 (53.3)	1.179	10 (4.3)	16 (6.8)	24 (10.2)	47 (20.0)	127 (54.0)	11 (4.7)	235 (53.3)	5.161
Disagree	122 (59.2)	84 (40.8)	206 (46.7)	0.278	8 (3.9)	22 (10.7)	28 (13.6)	37 (18.0)	97 (47.1)	14 (6.8)	206 (46.7)	0.397

*Means significant.

When asked about using AI simulators instead of patients in clinical rounds, opinions were nearly balanced, with 52.2% agreeing and 47.8% disagreeing. Agreement was higher among males (65.2%) compared to females (34.8%). Interestingly, academic year distribution showed that support was most concentrated in the 5th year (50.0%). A statistically significant difference was observed in this question (*p* = 0.030), suggesting meaningful variation by academic year.

In terms of learning preferences, 60.1% of students favored AI-generated personalized questions over traditional resources. This preference was more common among males (63.8%) than females (36.2%), with the largest share in the 5th year (50.9%). However, 39.9% still preferred traditional methods. Finally, regarding the idea that AI could replace traditional teaching methods, responses were mixed: 53.3% agreed while 46.7% disagreed. Male students showed slightly higher agreement (64.3%), and once again, 5th-year students formed the majority among those in favor (54.0%).

## Discussion

The present study demonstrated a high level of awareness of artificial intelligence (AI) among medical students; however, only a small proportion had received formal AI education or structured training. Students primarily relied on informal sources such as media, online platforms, and personal networks highlighting that media remains the dominant and often first point of contact for AI information. Similar findings were reported by AlZaabi et al. (2), Wood et al. (8), and Biagini (9), who found that medical students generally possess high awareness of AI but have limited formal training and practical exposure to its healthcare applications and insufficient curricular integration of AI (1).

The predominance of informal learning sources, including social media, online platforms, and peer networks, further highlights the gap between awareness and structured educational exposure. AlZaabi et al. (2) and Wood et al. (8) similarly observed that informal digital platforms were the primary source of AI-related information among medical trainees because AI-related topics remain insufficiently incorporated into undergraduate medical curricula. This reliance on informal learning sources highlights a potential educational gap, as the absence of structured instruction may result in fragmented understanding and misconceptions regarding the capabilities, limitations, and appropriate applications of AI in healthcare (2, 9).

AI literacy is increasingly recognized as an essential competency for future physicians. However, despite growing awareness and interest, structured AI education and curriculum framework remains limited in many medical schools and institutions worldwide. Consistent with the present findings, Wood et al. (8) and Tran et al. (5) reported that both students and faculty members expressed readiness for AI-related instruction, while curricula remain outdated and inadequate to equip students with AI competencies or prepare future clinicians for AI-driven healthcare systems (5, 9). These findings underscore the need for integrating AI concepts, applications, and ethical considerations into undergraduate medical education to better prepare future healthcare professionals.

A predominantly positive attitude toward AI was observed among the participants. This finding is consistent with studies conducted by Jha et al. (1) and Khater et al. (12), who reported favorable perceptions regarding the potential of AI to improve diagnostic accuracy, clinical decision-making, and healthcare efficiency. They found also strong enthusiasm among medical students and faculty for integrating AI tools into clinical and educational settings, provided that appropriate training and ethical safeguards are established. The consistency of these findings across geographic settings suggests that medical students globally are receptive to AI adoption and willing to integrate it into future practice (1, 12). Furthermore, the significant association between academic year and attitudes toward AI observed in the present study is comparable to findings reported by Tran et al. (5), who noted that educational exposure influences students’ perceptions of AI technologies they also showed that early exposure to technology and preclinical enthusiasm often correspond with more favorable attitudes before students confront the ethical and practical complexities present in clinical training. Similarly, Mehta et al. (10) observed differences in student engagement according to academic level, with students in earlier years demonstrating greater interest in AI due to their enthusiasm for emerging technologies and increased opportunities for early exposure. This finding may help explain the significant association between academic year and attitudes toward AI observed in the present study.

Participants recognized several potential benefits of AI in healthcare, particularly in diagnostic support, medical imaging interpretation, chronic disease monitoring, and precision medicine. Where most medical students believed that AI would be most widely utilized in clinical specialties especially image-based specialty that rely heavily on electronic health records, Digital screening, and Medical imaging analysis particularly pathology, radiology, and dermatology because of computer programs with artificial intelligence recognize imaging procedures and x-ray better than doctors and make fewer mistakes. Similar findings were reported by Sassis et al. (3) and AlZaabi et al. (2), who identified radiology, pathology, dermatology, and genomics as specialties most likely to benefit from AI implementation. This perception likely reflects the growing role of AI in medical imaging, pattern recognition, digital screening, and the analysis of large clinical datasets. Global evidence supports this expectation, as AI systems have demonstrated considerable accuracy in image interpretation and diagnostic support, making imaging-intensive specialties among the earliest adopters of AI technologies (2). However, their study reported lower expectations regarding AI applications in automated surgical procedures and primary care. Such differences may reflect variations in students’ awareness of emerging AI applications beyond diagnostic imaging, as well as perceived technical, financial, and infrastructural challenges associated with implementing AI in more complex clinical settings. Limited exposure to advanced AI technologies may also contribute to a narrower perception of AI’s potential role in surgical and primary care specialties (2). Participants identified chatbots, virtual simulations, interactive smartboards, and automated grading systems as the most preferred AI-based educational tools. These findings differ somewhat from those reported by Khater et al. (12) at Ain Shams University, where virtual patient learning (63.4%) and virtual reality simulation (59.3%) were the most utilized AI-supported educational approaches. In their study, interactive smartboards, automated examination grading systems, and personalized learning platforms were reported at comparable rates (47.8, 46.8, and 45.4%, respectively), whereas gamification tools were the least frequently preferred educational technology (21.2%) (12). The observed differences between the two studies may be attributed to variations in technological infrastructure, resource availability, institutional support, and students’ exposure to simulation-based and AI-enhanced learning environments.

Despite these positive perceptions, students expressed concerns regarding data privacy, ethical issues, accountability, algorithmic bias, diminished clinical reasoning, and reduced physician–patient interaction. Wood et al. (8) who emphasized the importance of educating future physicians about ethical and legal considerations associated with AI implementation in healthcare by incorporating structured ethics education into medical curricula to address issues such as algorithmic bias, data privacy, data misuse, and AI-assisted decision-making (9). Furthermore, several evidence suggested that students may overestimate the capabilities of AI while underestimating its limitations and potential risks. This underscores the need for comprehensive education on AI safety, governance, accountability, and the appropriate use of AI technologies in clinical practice (9).

Participants also identified barriers to AI adoption, including inadequate training opportunities, limited technological infrastructure, financial constraints, and insufficient institutional support. These findings are consistent with those reported by Additionally, the barriers to AI adoption identified in the present study—including insufficient training, limited technological and financial resources, and inadequate awareness—are consistent with findings reported by Khater et al. (12), who emphasized that successful AI integration requires not only curricular reform but also adequate institutional infrastructure, faculty development, and robust ethical frameworks. Similarly, Pinto dos Santos et al. (13) identified several challenges to AI implementation in medical education, including lack of awareness, limited curricular focus, and shortage of qualified faculty. Additional contributing factors include insufficient physician knowledge of AI, minimal undergraduate exposure, The lack of emphasis on artificial intelligence in current accreditation requirements, burdensome curriculum and frequent requests for new subjects and areas of study, the lack of faculty with the necessary expertise to teach this material, and the fact that medical schools and educators are still unsure of the precise emerging role of the physician in relation to AI (13).

In contrast, Yüzbaşıoğlu (14) reported that most students did not perceive lack of awareness as a major barrier to AI adoption and that may be because more technological adoption due to the revolution in the world of social media and the progress of these countries in education, scientific applications, and the devices available to doctors in the work environment and students in universities, which contributed to increasing their awareness of artificial intelligence and its great importance in various fields. Greater integration of technology into healthcare systems and medical education may contribute to higher levels of awareness and acceptance of AI among students in more technologically developed settings (14).

Regarding healthcare applications, students acknowledged the potential of AI to improve data management, clinical efficiency, and diagnostic accuracy. Similar observations were reported by Swed et al. (15) and Selamat et al. (16), who found strong support for AI-assisted healthcare services among medical students and healthcare professionals. However, perceptions regarding AI’s ability to reduce medical errors were less favorable in the present study than those reported by Swed et al. (15) in Syria and Selamat et al. (16) in Malaysia reported greater confidence in AI’s ability to reduce medical errors, with approximately 40% and two-thirds of participants, respectively, supporting this view.

An even higher level of agreement was reported by Syed and Al-Rawi (17) in Saudi Arabia, where 75% of participants believed that AI could contribute to reducing medical errors and greater confidence in AI’s potential to enhance patient safety. This perception may be explained by the growing recognition of machine learning algorithms as valuable tools for clinical decision support, data analysis, and diagnostic accuracy. By processing large volumes of clinical data and identifying relevant patterns, AI systems have the potential to reduce unwarranted variations in practice, improve efficiency, support diagnostic evaluation, and ultimately contribute to safer patient care (17).

Students demonstrated moderate willingness to use AI in practice, with most indicating occasional use. This contrasts with higher usage rates reported by Giavina-Bianchi et al. (18), reflecting greater infrastructure and AI integration in Brazilian healthcare settings which suggest variability in technological readiness across settings. Perceptions of AI’s impact on patient appointments were mixed; they found that less than one third expected a decrease, less than one quarter expected an increase, and less than one fifth reported no change while most of them reported they do not know. Conversely, Giavina-Bianchi et al. (18) found that 33% expected an increase, 6% a decrease, and 49% no change. Such variability reflects differences in health system digitization and expectations of AI efficiency. There are several factors to consider when evaluating how AI may influence the number of patient appointments. For instance, AI-driven technologies such as telemedicine platforms and virtual assistants have the potential to streamline administrative processes, improve access to care, and facilitate remote consultations, thereby increasing the number of appointments for patients. On the other hand, concerns about job displacement and the efficiency of AI-driven algorithms may lead to a reduction in appointments as healthcare providers adopt these technologies (18).

Most participants anticipated that AI would become a routine component of medical practice within the next decade. Expectations varied by gender and academic year, with male and more senior students expressing greater optimism about the timeline for AI integration. Comparable expectations were reported by AlZaabi et al. (2), who found that many healthcare trainees expected widespread AI integration into clinical practice within the coming years.

The study examining whether AI can be used as a tool in medical practice showed that more than half of participants disagreed with the use of AI tools in personalized medicine, mainly due to concerns that current regulations are insufficient to protect individuals’ health data, which may be vulnerable to hacking or misuse. However, the remaining participants supported AI use, recognizing its potential to assist physicians in clinical decision-making, diagnosis, treatment, and other aspects of care. Opinions regarding the impact of AI on future employment were divided. While some students anticipated potential job displacement because AI can do many tasks that humans currently do, often more quickly and accurately, others believed AI would complement rather than replace physicians as there is an important role of human expertise as there are some tasks that are resistant to automation and require strategic thinking and human judgment. Stewart et al. (19) similarly concluded that AI is more likely to augment healthcare professionals’ capabilities than replace them entirely. Additionally, findings from Syed and Al-Rawi (17) in Riyadh, Saudi Arabia, indicated that while some participants believed AI could replace physicians, the majority viewed it as a complementary tool that supports healthcare professionals.

AlZaabi et al. (2) also reported higher optimism, with 54% predicting new jobs and fewer concerns about job loss. These variations reflect differences in digital ecosystem maturity and trust in AI governance. These differing viewpoints underscore the need for proactive measures to mitigate potential workforce disruptions while harnessing the transformative potential of AI in healthcare (20, 21).

.Finally, students expressed strong support for AI-based assessment and personalized learning, believing that AI tools would provide more objective grading and more personalized feedback during clinical skills examinations instead of human standardized patients aligning with findings by Doumat et al. (22), who found that 54.7% of students supported AI-assisted question generation. It may be due to the lack of traditional questions and the difficulties that come on our way to get it although opinions remained divided regarding using AI simulators in clinical rounds and on replacement of traditional teaching methods.

Overall, the findings demonstrate a substantial gap between awareness of AI and formal educational preparation. Although attitudes toward AI were generally positive, concerns regarding ethics, privacy, accountability, and implementation barriers remain prominent. These findings highlight the need for structured AI curricula, faculty development programs, and institutional support to ensure that future physicians are adequately prepared for AI-enabled healthcare environments.

### Implications for practice

There is a clear need to integrate structured AI education into medical curricula, focusing on both technical skills and ethical considerations. Enhancing faculty training, improving infrastructure, and establishing clear guidelines will support effective AI adoption. These steps are essential to prepare future physicians to use AI safely while maintaining patient-centered care.

#### The limitation of the study

This study has several limitations. The findings are based on self-reported data, which may be subject to response bias and social desirability bias. In addition, the use of a non-probability convenience sampling technique and recruitment from a single medical school may limit the generalizability of the findings to medical students in other universities and geographical regions. The sample was also restricted to students from one institution, which may not fully represent the diversity of medical students across Egypt. Furthermore, the cross-sectional design precludes the establishment of causal relationships between knowledge, attitudes, and perceptions toward artificial intelligence (AI). Future multicenter studies using probability-based sampling methods, larger and more diverse samples, and longitudinal or experimental designs are recommended to validate these findings and provide deeper insights into the integration of AI in medical education.

## Conclusion

Medical students in this study demonstrated high awareness and generally positive attitudes toward artificial intelligence yet reported limited formal training and curriculum integration. They valued AI applications that enhance interactive learning, assessment, and clinical practice, while expressing concerns regarding ethics, reduced human interaction, and accountability. These findings highlight the need to incorporate structured AI education into medical curricula, emphasizing both technical competencies and ethical frameworks, to prepare future physicians for safe and effective AI adoption in healthcare.

### Recommendations

This study highlights the importance of enhancing medical students’ knowledge and understanding of artificial intelligence (AI) in healthcare. It recommends integrating AI into medical curricula while addressing ethical considerations such as data privacy and bias. The findings also emphasize the potential of online learning for AI education and suggest practical strategies, including expert workshops, collaborations with AI companies, professional development opportunities, and access to online resources to prepare students for technology-driven healthcare practice.

## Data Availability

The original contributions presented in the study are included in the article/supplementary material, further inquiries can be directed to the corresponding author.
